# Endometrial Perivascular Progenitor Cells and Uterus Regeneration

**DOI:** 10.3390/jpm11060477

**Published:** 2021-05-27

**Authors:** Shiyuan Li, Lijun Ding

**Affiliations:** 1 Center for Reproductive Medicine, The Affiliated Drum Tower Hospital of Nanjing University Medical School, Nanjing 210008, China; mf20350121@smail.nju.edu.cn; 2 Center for Molecular Reproductive Medicine, Nanjing University, Nanjing 210008, China; 3 Center for Clinical Stem Cell Research, The Affiliated Drum Tower Hospital of Nanjing University Medical School, Nanjing 210008, China; 4 MRC Center for Regenerative Medicine, University of Edinburgh, Edinburgh EH16 4UU, UK

**Keywords:** endometrial stem cell, perivascular cell, CD146 pericyte, CD34 adventitial cell, Asherman’s syndrome

## Abstract

Ovarian steroid-regulated cyclical regeneration of the endometrium is crucial for endometrial receptivity and embryo implantation, and it is dependent on the dynamic remodeling of the endometrial vasculature. Perivascular cells, including pericytes surrounding capillaries and microvessels and adventitial cells located in the outermost layer of large vessels, show properties of mesenchymal stem cells, and they are thus promising candidates for uterine regeneration. In this review, we discuss the structure and functions of the endometrial blood vasculature and their roles in endometrial regeneration, the main biomarkers and characteristics of perivascular cells in the endometrium, and stem cell-based angiogenetic therapy for Asherman’s syndrome.

## 1. Introduction

During a woman’s reproductive years, the endometrium dynamically undergoes around 400 cycles of morphological and functional changes, and the cyclic change of the endometrium is crucial for embryo implantation. The human endometrium comprises the upper two-thirds functional layer, which contains superficial epithelial cells and the underlying stroma, and the lower third basal layer. The functional layer uniquely goes through extensive proliferation and slough, while the basal layer does not shed during each menstrual cycle.

In the secretory phase, decidualization occurring in the endometrial stromal cells allows the maternal body to be immunotolerant to the allogenic embryo, and without pregnancy, the consequent menstruation enables the functional layer to be fully denuded and ready for regeneration, which only occurs in primates [1]. Thereafter, the functional layer grows from the basal layer and is repaired in response to the rebound of estrogen in the proliferative phase [2]. This process is regulated by ovarian steroids and accompanied by angiogenesis and vessel regression. Malformation of blood vessels is related to gynecological disorders, such as menoxenia and endometriosis, and can lead to failure in embryo implantation and infertility [3,4]. Hence, the endometrium is a tissue where physiological injury and scar-free repair recurrently occur and for which the basal layer is postulated as a place possessing stem/progenitor cells [5]. Once the uterus experiences severe trauma, such as curettage, cesarean section, hysteroscopic surgery, or infections that destroy the basal layer, fibroblasts are overactivated and secrete collagen, resulting in improper regeneration and formation of uterine scars and intrauterine adhesions (IUA) [6,7,8,9].

Supplementation of stem/progenitor cells to compensate for the debilitating regeneration of the endometrium is considered to shed a sliver of light for IUA patients. Among several stem/progenitor cells under discussion, perivascular cells, including pericytes surrounding the capillaries and microvessels, as well as adventitial cells located in the outermost layer of large vessels, have been showing phenotypes of mesenchymal stem cells (MSCs) and regenerating capacities [10].

This review introduces the development and structure of the endometrial vasculature, and examines how blood vessels vary in response to the cyclic change in ovarian steroids. The identity, characteristics, and different biomarkers of the perivascular cells and the associations between pericytes and adventitial cells are discussed. Furthermore, although with limited applications in both animal and clinical trials, the regenerating potential and underlying mechanisms of uterine repair based on perivascular cells in the treatment of severe Asherman’s syndrome (AS) or IUA are also discussed.

## 2. The Endometrial Vasculature

**Figure 1:**
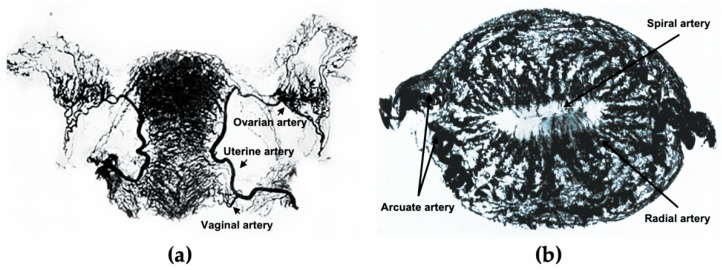
The uterine vasculature. (**a**) Arterial blood supply of the ovaries, fallopian tubes, and uterus. (**b**) Radiographs of the transverse slice of the uterus in the mid-secretory phase, showing its arterial vasculature. Figure 1a is edited from the figure by Norris, C.C., Clark, J.G., and Peters, D. *Gonorrhea in Women*. **1913**. Figure 1b is edited from the figure by Farrer-Brown, G., Beilby, J.O., and Tarbit, M.H. The Blood Supply of the Uterus. 1. Arterial Vasculature. *J. Obstet. Gynaecol. Br. Commonw.* **1970**, *77*, 673–681.

In the 10th week of pregnancy, bilateral Müllerian ducts fuse to form the uterus, and the endometrium completely differentiates from uterine mucosa by the 20th week [11]. The development of the uterus is accompanied by the formation of the uterine vasculature, which comes from the uterine, ovarian, and vaginal arteries (Figure 1a). The lateral branches emit anterior and posterior arcuate arteries, which then extend into the myometrium in the circumferential direction, nourishing two-thirds of the myometrium and divisions ending in capillary networks surrounding muscle fibers [12]. After entering the endometrium, the number of spiral vessels sharply decreases compared with the myometrium, with dynamic variations in tortuosity during different menstrual phases (Figure 1b) [12].

The formation of primitive blood vessels, also known as vasculogenesis, originates from endothelial progenitors called angioblasts, which are derived from mesodermal cells, differentiate into endothelial cells, and migrate and coalesce to form the early vascular plexus [13]. The developing needs and microenvironment call for organ-specific endothelial specialization and mural cell coverage to deliver nutrients and gases, absorb and filter fluids, assist metabolic processes, and participate in the regulation of local immune cells [14]. Thereafter, blood vessels undergo remodeling and expansion and form the vasculature network [15]. The process of new blood vessels growing out from the existing vasculature is known as angiogenesis, which occurs in both physiological development process and pathological conditions [16]. The vital role of angiogenesis has been widely studied during embryogenesis, where the establishment of circulation is no doubt a necessity for fetal development, and pathological processes such as inflammation and malignant tumor growth where the formation of new vessels both provides nourishing effects and supports immune responses [17,18]. Despite considerable research exploring the processes of uterine development and fetal blood vessel formation, including both vasculogenesis and angiogenesis, the role of uterine-specific vasculature formation in uterine development remains unclear.

Variations in blood vessels rarely occur under physiological conditions in adults, except for the reproductive system. During the secretory phase of the menstrual cycle, the uterus is mainly supplied by the uterine artery, while a greater proportion of the uterus around the uterine horns is supplied by the ovarian artery during the proliferative phase [19]. Periodic changes in the ratio of progesterone and estrogen from ovaries regulate the blood flow of uterus-supplying arteries, and the distribution of ovarian steroids delivered by peri-uterine vessels varies from that by systemic vessels, which shows the mutually reinforcing relationship between blood vessels and the reproductive apparatus [19]. Furthermore, the morphology of the blood vessels dynamically changes with the menstrual cycle. Under physiological conditions, straight arterioles branch out of the arcuate arteries that are located in the uterine myometrium, which contain endothelial cells, surrounding smooth muscle cells (SMCs), and perivascular cells. The morphology of arterioles in the functional layer gradually varies from straight to spiral as the proliferative phase progresses, and the vessel wall becomes thinner as the arterioles pass through, leaving only vascular endothelial cells when these reach the subepithelial luminal surface [20]. Endothelial cells that are not connected to SMCs or perivascular cells in the functionalis are susceptible to apoptosis when angiogenic factors are withdrawn, while the endothelial cells in the basalis and connected with pericytes remain stable [20]. Factors including the VEGF family, hypoxia-inducible factor, angiopoietins, and fibroblast growth factor family are involved in the process of endometrial angiogenesis, regulating the proliferation and migration of endothelial cells [20].

Three processes that angiogenesis prominently participates in are: (1) post-menstrual repair after vascular bed rupture, (2) endometrial growth during proliferative phase when the thickness of the endometrium increases by up to fourfold, and (3) the significant elongation and coiling of spiral arterioles during the secretory phase [21]. Four forms of angiogenesis include sprouting, intussusception, vessel elongation, and circulating endothelial progenitor cells that incorporate into growing vessels [22,23,24]. In spite of the initial and conventional efforts exerted into studies on vessel sprouting, it has been reported that non-sprouting angiogenesis plays a major role in the cyclic generation of the uterus. In particular, vessel elongation plays a major role in the mid–late proliferative phase, while the angiogenic pattern in the early–mid secretory phase tends to be intussusception [25].

Angiogenesis is relatively active in the proliferative phase, and mitotic activity reflected by nuclear DNA synthesis is more intense in the functionalis than in the basalis layer throughout the proliferative phase [26]. Interestingly, the rate of proliferation of endothelial cells does not significantly vary during the cycle and is still maintained in the late-secretory and menstrual phase, when there is no significant endometrial growth [27,28], although the lack of a proliferation peak may probably be due to inter-individual variations [26]. To determine the correlation between steroid changes and endometrial angiogenesis, a model was created by Nayak and Brenner, in which rhesus macaques were ovariectomized and supplemented with estradiol and progesterone to mimic the natural menstrual cycle [29]. Proliferation of endothelial cells, as detected by Ki-67 and BrdU, increased 6-fold at 8–10 days after the withdrawal of progesterone, reached the bottom at menses, and remained steady in other stages. This result suggests that angiogenesis is an estrogen-dependent process that mainly occurs in the mid-proliferative phase and is in line with VEGF expression in the endometrial stroma at this phase [29]. Given that the mechanism of angiogenesis in the endometrium is rather complex, measuring methods in addition to the proliferation of endothelial cells, such as laser Doppler imaging and visualization by pigment particle perfusion, should be introduced to study the growth pattern of blood vessels [30].

In addition to angiogenesis, vessel maturation is another process that is required for regeneration after endometrial shedding. Mural cells, including vascular smooth muscle cells and pericytes, proliferate under the control of progesterone during the secretory phase, and wrap around endothelial cells, regulating angiogenesis and coordinating the function of blood vessels [31]. In the late-secretory phase, perivascular cells form a thick layer and surround the spiral arterioles, not only resembling the decidual cells, but also maintaining the capacity to interact with endothelial cells [32].

Aberrations in angiogenesis are associated with gynecological clinical consequences. The proliferation rate of endothelial cells instead of endometrial stromal cells, glandular cells, and surface epithelial cells has been reported to be higher in patients with menorrhagia [33]. It has also been reported that intense vascularization exists in endometriosis implants, with a significantly higher secretion of VEGF in peritoneal fluids in the proliferative phase of the menstrual cycle [34,35]. On the other hand, in severe IUA patients with a narrow uterine cavity, fibrotic scarring, vague uterine horns, and absence of glands, blood flow is poor in the pale endometrial tissues, where angiogenesis is completely blocked [36]. Therefore, the dynamic balance between promotion and inhibition of blood vessel growth is inseparable from maintenance of the normal function of the endometrium.

## 3. Endometrial Perivascular Cells as Native MSCs

The human endometrium can grow 4–6 mm within 5–6 days after menstrual shedding, indicating its remarkable regenerative ability and the participation of stem/progenitor cells to support self-maintenance [37,38]. On days 2–3 of the menstrual cycle, the endometrium is rapidly covered within 48 h by surface epithelia that originate from two major sources, namely, the exposed stump of basal glands and the remaining surface epithelium bordering the denuded areas [39]. Therefore, the basal layer has been considered as the place where stem/progenitor cells reside and contribute to the construction of functional layers [40]. Progenitor cells in the endometrium respond to hormonal changes and regenerate the endometrium and vasculature in each menstrual cycle, as well as support stromal decidualization during embryo implantation [41]

**Figure 2:**
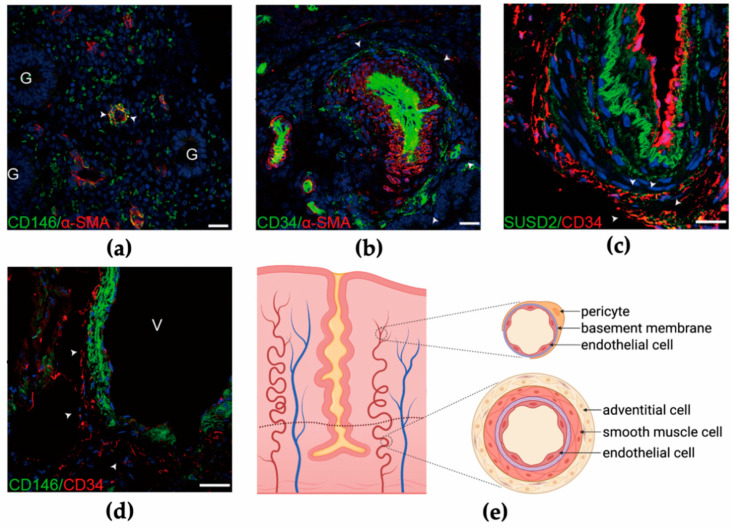
Perivascular cells in the human endometrium. (**a**) Co-staining of CD146 (green) and α-SMA (red) in human endometrial functionalis. G, glandular epithelial cell. (**b**) Cells expressing α-SMA in the tunica media are surrounded by CD34^+^ adventitial cells in human endometrial basalis. (**c**) Part of the CD34^+^ adventitial cells are co-stained with SUSD2. (**d**) CD34^+^ adventitial cells enclose CD146^+^ pericytes in large vessels in the human endometrium. V, vessel. (**e**) Schematic showing two groups of perivascular cells in the endometrium. Pericytes surrounding endothelial cells are located in microvessels in both functionalis and basalis, while adventitial cells reside in the outermost layer of large vessels, mainly in endometrial basalis. Figure 2a–d from Zhu, X., Yu, F., Yan, G., Hu, Y., Sun, H., and Ding, L. Human endometrial perivascular stem cells exhibit a limited potential to regenerate endometrium after xenotransplantation. *Hum. Reprod.* **2020**.

Prianishnikov postulated in the 1970s that stem cells are located in the glandular epithelia of endometrial basalis, and differentiated daughter cells gradually mature with the acquisition of hormonal receptiveness [42]. However, due to the difficulty in culturing epithelial cells in vitro, it is difficult to determine whether these possess stem cell properties and the capacity to differentiate into other kinds of endometrial cells [38]. It was not until 2004 that clonogenicity of a small group of stromal (1.25%) and epithelial cells (0.22%) was confirmed, indicating the precursor role in the endometrium [43]. The epithelial clones from the human endometrium can differentiate into gland-like structures in three-dimensional culture, and the stromal clones are multipotent, expressing MSC markers [44]. Subsequently, two markers, CD146 (or MCAM) and platelet-derived growth factor-receptor β (PDGF-Rβ), have been identified from the colony-forming endometrial stromal cells, which exhibit MSC properties [45]. Crisan et al. have shown that both CD146 and PDGF-Rβ are ubiquitously expressed on pericytes surrounding endothelial cells in the capillaries of all human organs, and the co-expression of NG2 and deficient expression of endothelial markers (CD144, CD31, CD34, vWF, and UEA-1) were further confirmed by immunodetection [46]. CD146^+^ pericytes mainly reside in capillaries and microvessels in both the functionalis and basalis of the endometrium (Figure 2a) [47]. Moreover, higher clonogenicity was observed in CD146^+^PDGF-Rβ^+^ cells expressing SUSD2 (or W5C5), which in vivo constitute endometrial stromal tissues [48].

Pericytes, also known as mural cells or Rouget cells, encircle the capillaries and microvessels and play a role in angiogenesis and in regulating blood pressure using their contractive behavior [49]. Pericytes are responsible for the formation of fibroblast-like cells and the deposition of the extracellular matrix, which hinders axonal regeneration in spinal cord injuries [50]. Purified as CD146^+^CD34^−^CD45^−^CD56^−^ cells by flow cytometry in multiple organs, pericytes are contemplated as the origin of MSCs, in view of several phenotypes and expressed genes shared by both kinds of cells [51]. First, pericytes possess similar adherence and morphology as MSCs and can be cultured into fibroblast-like cells in vitro. Second, both these express the classic MSC markers CD29, CD44, CD105, CD73, and CD90, and they do not express markers of hematopoietic stem cells such as CD45 and human leukocyte antigen-antigen D-related (HLA-DR). Third, CD146^+^ pericytes can be induced to differentiate into multiple mesodermal lineages, including adipogenic cells, osteogenic cells, and neural-like cells. Particularly in the endometrium, CD146^+^PDGF-Rβ^+^ cells are closely related to stromal fibroblasts, which were identified by PCA and hierarchical clustering and are capable of differentiating into stromal fibroblasts in vivo, suggesting that these belong to the same lineage [52].

Subsequent research has determined that pericytes are not the only origin of MSCs. CD34^+^CD146^−^CD31^−^CD45^−^ adventitial cells have been sorted by flow cytometry from multiple organs as another cluster of cells that show characteristics of mesenchymal stem-like cells [51]. Adventitial cells, which are located in the outermost layer of large vessels, contribute to the maintenance of blood vessel structure and stiffness, participate in the regulation of vasculature hemostasis, and influence the function of the tunica intima and media during pathological processes [53,54]. The tunica adventitia is also presumed as a niche for stem/progenitor cells. Adventitial cells that positively stain for Sca-1 and CD34 have been reported to directly differentiate into smooth muscle cells in atherosclerotic lesions and into osteoblast-like cells, contributing to arterial calcification in chronic kidney diseases [55,56]. Besides, CD34^+^ adventitial cells from vessels with diameters >50 μm in white adipose tissue and that do not express endothelial markers such as CD31 and CD144 have been shown to be capable of clonogenicity, self-renewal, and multilineage mesodermal differentiation [57]. Interestingly, CD34^+^ adventitial cells express pericyte markers such as CD146, PDGFR-β, α-SMA, and NG2 after exposure to angiopoietin-2, suggesting that adventitial cells are the precursors of pericytes [57]. Recently, CD34^+^CD146^−^CD45^−^CD56^−^CD144^−^ adventitial cells have also been proven for the first time to exist in human endometrial tissues and are located in the outermost layer of large vessels, particularly the basal layer (Figure 2b), with an abundance nearly 40 times higher than that of pericytes [47]. Furthermore, SUSD2, another marker of perivascular cells that possesses MSC properties, is expressed mostly in the tunica media of vessels and in only a small proportion of CD34^+^ adventitial cells (Figure 2c). After inducing culture, both CD34^+^ adventitial cells and CD146^+^ pericytes derived from the human endometrium, could differentiate into endometrial stromal-like cells, while the expression levels of vimentin and CD13 significantly increased in adventitial cells [47].

Collectively, the blood vessel wall harboring perivascular cells is considered as a place that gives rise to multipotent cells. Pericytes encircling endothelial cells in capillaries and microvessels in both the functionalis and basalis, as well as adventitial cells that reside in larger vessels in the basalis, are the two precursors that exhibit MSC phenotypes and have the potential to develop in the human endometrium (Figure 2d,e). Although protocols have been established for isolating perivascular cells from blood vessels, a high demand for standardized selection criteria for perivascular cells for regenerative medicine remains. Meanwhile, the hierarchical relationship between pericytes and adventitial cells requires further investigation. In several intraluminal or perivascular lesions in the carotid and coronary arteries, migration of adventitial fibroblasts into the neointima in response to vascular injury has been observed [58,59,60]. Therefore, it is important to determine whether adventitial cells from the outside layer play a predominant role in vessel regeneration and remodeling such as that in menstruation, where the integrity of endometrial vessels endures spontaneous compromise and restoration. Other putative cell groups showing progenitor roles and potentially responsible for tissue regeneration include stromal and epithelial Lgr5^+^ cells, side population (SP), MSCs from menstrual blood, and migrated bone marrow cells [61,62,63,64]. Despite all of the candidates that have arisen, the representative bio-markers and the exact location of endometrial stem cells have not been fully established.

## 4. Perivascular Cell-Based Angiogenetic Therapy for Asherman’s Syndrome (AS)

AS is characterized by fibrotic stroma and an inert endometrium that is unresponsive to steroid hormones, and leads to clinical manifestations, including menstrual abnormalities, pelvic pain, recurrent miscarriage, and infertility [65]. IUA classification formulated by the American Fertility Society (AFS) in 1988 and the European Society of Gynecological Endoscopy (ESGE) in 1995 is now commonly used in clinical practice in China. Based on the features observed through hysteroscopy and the patient’s menstrual pattern, IUA was classified into mild, moderate, and severe by the AFS [66], and the extent of adhesions was more accurately sorted using the ESGR criteria [67]. The classification result is closely related to the reproductive outcomes of IUA patients [68].

Surgical techniques using hysteroscopic adhesiolysis have been extensively developed and applied to AS treatment to remove adherent lesions and restore uterine patency, and they are recommended for patients with fertility requirements. However, treatment of severe IUA is required for the restoration of both patency of the uterine cavity and endometrial function, which include the regeneration of parenchymal cells and neovascularization. Given that the viability and function of the endometrium could not be simply overturned and resumed, pregnancy prognosis involved with spontaneous miscarriage rate and placenta abnormality has not been well improved in women who received AS surgical treatment [69]. The conception rate of severe IUA patients (25%) remains significantly lower than that of mild and moderate IUA patients (60.7% and 53.4%, respectively) after adhesiolysis [70], while stem cell transplantation is considered an alternative for endometrial regeneration and fibrotic area repair to allow embryo implantation in severe cases [71].

MSCs are ideal cells with application prospects for tissue regeneration because these can be isolated from various organs using mature techniques, with lower tumorigenic potential than embryonic stem cells, and their immunomodulatory roles enable MSCs to be tolerated by the transplant recipient [72]. Thus far, MSCs derived from bone marrow, adipose tissue, and umbilical cord have been applied through intrauterine or tail vein injection into rat models with AS, which were proved to induce endometrial proliferation, reduce fibrotic areas and inflammation, as well as promote angiogenesis and enhance pregnancy outcome [73,74,75].

Perivascular cells are postulated to be the precursors of tissue-specific MSCs [76], and their progenitor role in tissue development and regeneration has been investigated in several diseases, such as incisor injury, ischemic stroke, and calvarial bone defect [77,78,79]. Therefore, the regenerating capacity lying in the perivascular region for AS treatment is worthy of further discussion.

The regenerating functions exerted by perivascular cells mainly lie in their aforementioned intrinsic progenitor identity. CM-Dil-labeled MSCs isolated from the umbilical cord and intraperitoneally injected into AS rats were found to migrate to the stromal layer and begin to differentiate into epithelial cells, although the few cytokeratin-expressing cells observed were located in the stroma rather than epithelium, vascular endothelial cells, and cells with estrogen receptors [80]. Although suggested as the source of supplementary cells in injured areas, MSCs are gaining more attention for their paracrine effect. After injecting stained perivascular cells through the mouse tail, cells migrated to the injured uteri, while the number of remaining cells sharply decreased within seven days, indicating that the regenerative effects were mainly induced in a paracrine manner [81]. Secreted bioactive cytokines or growth factors are known to exhibit properties such as anti-apoptosis and anti-fibrosis, promote angiogenesis, trigger tissue-specific progenitors, and modulate immune responses [82]. However, the exact content of secretomes in perivascular cells undergoing uterine regeneration and their corresponding functions remain unclear.

Furthermore, considering the original role of pericytes in vessel walls, these also stimulate angiogenesis and promote the stabilization and maturation of vessels, which is essential to endometrial growth [83]. Oct4 expressed by perivascular cells may essentially contribute to the angiogenetic capacity, and its specific knockout hinders the migration of perivascular cells and endothelial cells and intensifies vascular leakage [84]. In addition, through the paracrine effect, pericytes interact with endothelial cells and promote vascular homeostasis by secreting factors included in pathways such as VEGF, PDGF-B, TGF-β, and Ang signaling [85]. In another AS mouse model treated with CD146^+^SSEA-4^+^ pericytes from human umbilical cords, increased expression of proangiogenic factors, including HIF1α, VEGF, ANG-1, and TIE2, and the prerequisite role of angiogenesis in endometrial regeneration were determined [81]. It has been documented that CD146^+^ pericytes from the human endometrium could be utilized in uterine regeneration in rat models that underwent 1.5 cm × 0.5 cm full-thickness uterine resection, with a focus on expounding the capacity of in vitro angiogenesis and in vivo neovascularization by pericytes, which is probably induced by secretion of CYR61 [86]. Moreover, when induced on Matrigel for tube formation analysis, CD34^+^ adventitial cells formed branches at an earlier stage than CD146^+^ pericytes, and later developed a network loop structure that positively stained for CD31 and vWF [47]. Nevertheless, whether CD34^+^ adventitial cells embody a more compelling angiogenetic capacity in vivo in injured tissues, particularly in an AS uterus, and whether perivascular cells contribute to tissue regeneration through the angiogenetic process remains unclear.

Additionally, transcriptomic analysis of CD146^+^PDGF-Rβ^+^ endometrial cells has revealed the upregulated expression of genes related to immunomodulatory functions and responses to hypoxia, inflammation, and proteolysis, thereby supporting the role of pericytes in endometrial changes during menstruation [52]. Because undifferentiated MSCs only express MHC class I, allogeneic MSCs are able to escape from T cell surveillance when these are applied to injured areas [87]. Other repairing mechanisms may include fast re-epithelialization in damaged uterus, as reported by Yin et al.; CD34^+^KLF4^+^ endometrial stromal cells detected by lineage tracing are responsible for epithelial growth 72 to 96 h after withdrawal of progesterone [88].

Collectively, perivascular cells participate in endometrial regeneration potentially by combining the advantages of their stem cell identity, angiogenetic ability, and immunoregulatory roles. However, how these potential repair mechanisms are orchestrated and what are the more prominent therapeutic effects underlying perivascular cells compared with ordinary MSCs still requires further investigation.

Although perivascular cells have not yet been utilized in clinical practice, the therapeutic effect of stem cells has already been reported in IUA patients several times. A meta-analysis on AS patients who had previously failed in surgical or hormone replacement treatment and subsequently received stem cell-based therapy revealed overall benefits, including improved menstruation, thickened endometrium, and successful pregnancy [89]. The recovery of endometrial thickness and vascularity as well as gravidity through IVF-ET were achieved in a severe IUA patient who received an intrauterine injection of autologous bone marrow stem cells [90]. Moreover, in a phase I clinical trial, the enhancement of endometrial cell proliferation and angiogenesis, and regaining of endometrial thickness and blood flow were, respectively, observed by histology and transvaginal sonography in IUA patients after transplanting 1 × 10^7^ allogenic umbilical cord MSCs, and 38.5% patients became pregnant within a 30-month follow-up. Safety of the treatment was also assessed, with no surgical complications, and systematic and local inflammation found during the whole research process [91]. Therefore, stem/progenitor cell-based therapy provides insights into the direct or indirect promotion of endometrial regeneration and functional restoration. Furthermore, although safety assessment of certain kinds of perivascular cells, including artery or vein perivascular cells from umbilical cord, has shown no tumorigenesis in immunodeficient mice, perivascular cells have hardly been applied in clinical settings so far [92]. The range of the number of perivascular cells transplanted, and the safety and toxicological data are supposed to arouse close attention as the follow-up trials unfold.

Usually, MSCs are delivered through systematic administration or local injection. However, a therapeutic response is hard to be satisfactorily achieved due to either insufficient accumulation in target tissues or risks following invasive operations. Herein, biomaterial scaffolds embedded with therapeutic cells are able to improve cell localization, and reduce tissue damage, and are hypothesized to provide a microenvironment for MSCs and influence their behavior [93,94]. Bone marrow MSCs loading on collagen scaffolds have been reported for application to damaged uterine tissues, which partially differentiated into cells that positively stain for desmin and vimentin, which are known markers of stromal cells, and the expression of growth factors (bFGF, IGF-1, TGF-β1, and VEGF) was detected within two weeks after transplantation, indicating both direct differentiation and secretory effect of MSCs [95]. In addition, an auto-crosslinked hyaluronic acid gel loaded with umbilical cord MSCs in AS rhesus monkey models was found to help uterine regeneration by preventing adhesion as a physical barrier and providing an attachment site for MSCs [96]. Moreover, three-dimensional (3D) printed hydrogels are another biomaterial in AS treatment, providing a spatial and physiological environment for MSC planting [97]. Despite all of the aforementioned findings, issues such as a combined system of perivascular cells and biomaterials in AS models and a more precise and noninvasive way to deliver the system warrant further investigation.

## 5. Conclusions

In conclusion, the progenitor role of perivascular cells derived from human endometrium and a stem cell-based therapy for AS have been investigated in both murine model studies and clinical trials. Nevertheless, several crucial questions have yet to be solved. First, the relationship between human endometrial vasculature and uterine development under physiological conditions remains unknown. The synchronous development of uterus and blood vessels is to be further revealed by combining the means of embryo anatomy, multi-omics analysis, and single-cell sequencing. Second, the role of perivascular cells during the processes of uterine development and cyclic endometrial regeneration needs to be further explored by in vivo cell lineage tracing. Third, by combining with tissue engineering, perivascular cells are promising candidates for tissue repair under pathological conditions, while better seed cells and improved delivery methods need to be explored. Moreover, the mechanisms behind how the precursor cells mobilize the remaining local cells and how they induce microenvironmental changes to allow themselves to colonize remain unclear.
